# Shifting self-perceptions of ageing: differential effects of value priorities on self-perceptions of ageing beyond age stereotypes

**DOI:** 10.1007/s10433-020-00578-3

**Published:** 2020-08-11

**Authors:** Anne Blawert, Susanne Wurm

**Affiliations:** Department of Preventive Research and Social Medicine, Institute of Community Medicine, University Medicine Greifswald, Walther-Rathenau-Str. 48, 17487 Greifswald, Germany

**Keywords:** Personal values, Personality, Value priority, Self-perceptions of ageing, Views on ageing

## Abstract

Self-perceptions of ageing (SPA) are important predictors of health in later life. However, research on antecedents of SPA other than age stereotypes is scarce. To address this gap, this study investigates the impact of personal value priorities beyond age stereotypes on SPA. Can values as the motivational basis of attitudes and evaluations predict gain- and loss-related SPA? To answer this question, we conducted multiple regression analyses of longitudinal data from two waves (2008, 2011) of the German Ageing Survey (DEAS; *N* = 6089, age range in 2008: 40–93 years). Gain- and loss-related SPA as well as age stereotypes were assessed with two AgeCog scales and personal values with the 21-item Portrait Values Questionnaire. Results indicate that value priorities relate to SPA longitudinally in domain-specific ways: People with a value priority of openness to change and self-transcendence reported more gain-related SPA at follow-up, whereas those who prioritized conservation reported less gain-related SPA. In the domain of loss-related SPA, those people with a value priority of self-enhancement reported more and those prioritizing self-transcendence reported less loss-related SPA at follow-up. These results complement and extend recent findings on the role of personality for SPA. They suggest that whether people focus on the gains or losses that occur with age, whether they perceive ageing as a threat or chance, is not only shaped by their age stereotypes, but also by what they find important—their values.

## Introduction

When you think about what is important to you, what springs to mind? Do you think you will still be able to reach your cherished goals when you are growing older? What people think about their own ageing, so-called self-perceptions of ageing (SPA) have been shown to predict a vast variety of outcomes such as health, well-being and even mortality (for an overview see e.g. Westerhof and Wurm 2018; Wurm et al. 2017). So far, only a few studies have examined antecedents of SPA (Bryant et al. 2016). Since the impact of SPA on health and longevity is well-evidenced, the time has now come to understand better how these self-perceptions themselves are formed. Whereas several studies pointed to the role of societally shaped age stereotypes for SPA (e.g. Levy 2003; Rothermund and Brandtstädter 2003), personal characteristics are just beginning to receive attention. Recently, some studies have started to investigate the role of personality for SPA. However, these studies mostly focused on personality traits based on the Five-Factor Model (Costa and McCrae 1992). Other aspects of personality, such as personal values, have not yet been considered despite values being potentially better suited than traits to predict cognitively based outcomes like SPA (Roccas et al. 2002). Thus, the role of personal values for SPA is still an open question and therefore addressed in the present study. The results will add to a more comprehensive understanding of the role of different aspects of personality in the context of SPA.

### The role of age stereotypes for SPA

“Old people are senile and inactive”–“old people are wise and share their life experience”. These generic statements are examples of typical stereotypes of older persons. So-called age stereotypes are defined as socially shared beliefs about older people as a group as well as the process of ageing (Wurm et al. 2017). Age stereotypes have consequences for self-views: Becca Levy’s Stereotype Embodiment Theory (Levy 2009) posits that societal age stereotypes are encountered from childhood onwards (e.g. the loving grandma, the forgetful, grumpy old man) and become internalized throughout the life span. At first, societal age stereotypes impact personal age stereotypes directed at “the elderly”; then, with increasing age, these “other”-stereotypes become “self”-stereotypes when they are applied to oneself, thus turning into SPA. Finally, these SPA can develop into a self-fulfilling prophecy as people with negative SPA experience more negative outcomes with ageing (Wurm et al. 2013). Substantial research has provided both cross-sectional and longitudinal evidence for the importance of societally shaped age stereotypes for SPA. However, SPA cannot be equated with age stereotypes; self-perceptions are more strongly characterized by personal experiences, individual biographies and personality (Wurm et al. 2017).

### The potential impact of values on SPA

Accumulating evidence points to meaningful longitudinal associations of personality and SPA for time periods up to 20 years, where personality traits seems to shape SPA rather than being shaped by them (Kornadt et al. 2019a). However, studies mostly focused on the “Big Five” traits of openness to experience, conscientiousness, extraversion, agreeableness and neuroticism. For example, Rupprecht et al. (2019) found conscientiousness to predict awareness of age-related gains longitudinally. Other studies found higher extraversion and agreeableness to predict lower perception of age-related losses (Bryant et al. 2016), whereas age-related growth was associated with higher extraversion, openness, agreeableness and conscientiousness (Shenkin et al. 2014). These results indicate that the trait-aspect of personality does contribute to SPA. Nevertheless, it is important to investigate the role of personal value priorities as well, as these two components of personality are associated with each other yet are not the same (e.g. Kandler et al. 2014); one can be open (trait) without finding openness important (value) and a person can value altruism highly without being very altruistic. Because of that, both values and traits are differentially suited for prediction of behaviour and other outcomes (Roccas et al. 2002).

Values are defined as guiding principles in people’s lives and express inherently desirable end-states that individuals try to reach. Schwartz’s well-known theory of basic human values (Schwartz 1992; Schwartz and Bilsky 1987) distinguishes four higher-order values, two of which express a focus on the self (agentic values: openness to change and self-enhancement) and two that express a focus on others (communal values: conservation and self-transcendence). The *openness to change* value emphasizes independent thoughts, actions and feelings as well as curiosity and readiness for new experiences. It represents pursuit of self-interest and independence of other people. The *self*-*enhancement* value emphasizes the pursuit of socially acknowledged success and respect as well as dominance over others. It represents a desire for high social status. The *conservation* value emphasizes self-restriction, order, preservation of the past and resistance to change. It represents how much a person believes people in general should adhere to social norms. Lastly, the *self*-*transcendence* value stresses a concern for the welfare of others and the world. It focuses on how a person wants to treat others and believes people and nature should be treated. It is an inherent feature of value theory that what drives behaviour and attitudes in an individual is not the absolute, but *relative* importance of a single value compared to the other values (Schwartz 2012)—the more important or self-central a value is in relation to the other values, the more it relates to an outcome (Gebauer et al. 2013). Following Schwartz and Rubel (2005), we refer to this relative importance of values as value priority.

Values serve as central criteria of evaluation of other people as well as the self and self-related issues (Schwartz 1992). Value priority directs attention to and interpretation of objective states (Schwartz et al. 2000); age is such an objective state and its evaluation and interpretation should then in part depend on the values that are most essential to a person. For example, an older person for whom openness to change is especially important might feel excited about an invitation to travel with other seniors and meet new people, whereas a person for whom conservation is most important might decline such an opportunity.

### Values, age stereotypes and SPA: a domain-specific approach

So far, associations between values and perceptions of ageing have predominantly been examined on a cross-cultural, societal level. A common belief is that individualistic Western cultures have more negative age stereotypes because of their values of youth-orientation, whereas collectivistic Eastern cultures have more positive age stereotypes due to values of filial piety and respect for elders (Löckenhoff et al. 2009). Building on these findings, Zhang et al. (2016) compared the impact of cultural versus personal values on individual age stereotypes, and found communal, “other-oriented” personal values to be significantly associated with individual age stereotypes. This suggests that values do play a role in the context of views on ageing. However, we are not aware of any study to date that examines the role of personal values for perceptions of the *own* ageing process. Yet if communal personal values are associated with views of others’ ageing, we assume agentic values in particular to be related to views of one’s own age and ageing, as agentic values focus on the self and interpretation of self-related issues. Since personality traits have been shown to provide additional explanatory power beyond age stereotypes cross-sectionally (Emile et al. 2014) and longitudinally (Levy 2008), we expect personal values to predict SPA beyond the impact of age stereotypes as well.

It is well established that age stereotypes are multi-directional and domain-specific, which means that people can simultaneously hold positive and negative age stereotypes in different life domains such as family (e.g. keeper of traditions) and cognition (e.g. inevitable decline); this also applies to SPA (e.g. Kornadt and Rothermund 2015). Thus, studies should examine differential effects of predictors for different domains of SPA (Spuling et al. 2019).

In the present study, we investigated the differential effects of personal value priorities in a loss- and a gain-related domain of SPA: The gain-related domain is that of ongoing psychological development. This SPA facet represents perceptions of ageing as a time of new plans and activities, of new skills and ideas. The loss-related domain is perception of age-related social losses. This facet does not refer to actual losses of relationships with close others, but to perceptions of social status loss such as being less respected, less needed and more socially isolated and lonely. We expected that each value priority uniquely predicts gain- and loss-related SPA.

#### Openness to change

The openness to change value is quite strongly related to the Big Five trait openness to experience (Fischer and Boer 2015; Parks-Leduc et al. 2015) which predicts more positive global (Emile et al. 2014) and, more specifically, gain-, but not loss-related SPA in longitudinal analyses (Bryant et al. 2016; Shenkin et al. 2014). Furthermore, Schwartz (2012) links this value to the promotion of gain-related goals as well as self-expansion and growth: A person who values new ideas and expressing the self should generally be more prone to perceiving opportunities to pursue this value even in old age or when confronted with age-related limitations.

#### Self-enhancement

A person valuing self-enhancement desires a high social status and strives for the respect and admiration of others. These aspects are negatively reflected in the SPA facet of ageing as associated with social losses. Ageing is often accompanied by loss of social status in certain areas: in midlife, children leave home and lead independent lives, thus no longer requiring parental care; with retirement, working life as a source for achievement and exertion of power vanishes. We assumed that a person for whom social status is relatively important would perceive these age-related changes in status as a threat to his or her cherished values. Furthermore, prioritizing self-enhancement and especially power is associated with higher worrying about self-related issues (Schwartz et al. 2000) and lower wellbeing (Sortheix and Schwartz 2017), which might also foster a loss-related view on ageing.

#### Conservation

Prioritizing stability, conformity and tradition implies resistance to change and a focus on preserving the past rather than looking for new experiences (Schwartz 2012). We supposed that this focus towards maintenance rather than development impedes gain-related SPA, that is, viewing ageing as a time of new plans and experiences: people who prioritize the value conservation might notice new opportunities, yet reject them.

#### Self-transcendence

Self-transcendence is an important component of wisdom (Curnow 1999) and has been theorized as the ultimate stage of human development and maturation, termed gerotranscendence (Tornstam 1994). As such, it can be seen as a more holistic view on life that emphasizes age-related change and development in a positive way (Tornstam 1997). The self-transcendence value is also related to the Big Five trait agreeableness (Fischer and Boer 2015; Parks-Leduc et al. 2015), which predicts lower perception of age-related losses over time (Bryant et al. 2016; Loi et al. 2015), and more perception of age-related gains in recent studies (Shenkin et al. 2014). Higher priority of self-transcendence is also associated with less worrying about the self (Schwartz et al. 2000), which could be reflected in more gain-related SPA. Therefore, we expected self-transcendence value priority to predict the perception of more age-related gains and less age-related losses at follow-up.

In short, the present study investigated these four hypotheses:

##### **H1**

Prioritizing openness to change predicts more gain-related SPA at follow-up, but not loss-related SPA at follow-up.

##### **H2**

Prioritizing self-enhancement predicts more loss-related SPA at follow-up, but not gain-related SPA at follow-up.

##### **H3**

Prioritizing conservation predicts less gain-related SPA at follow-up, but not loss-related SPA at follow-up.

##### **H4**

Prioritizing self-transcendence predicts more gain-related SPA and less loss-related SPA at follow-up.

## Methods

### Sample

Data was derived from the German Ageing Survey (DEAS; Klaus et al. (2017)), a representative, register-based, cohort-sequential longitudinal study of community-dwelling persons aged 40–85 that started in 1996. The analyses in this paper are based on the data of 6089 participants aged 40 and older that participated in the computer-assisted personal interviews (CAPI) and self-report questionnaire in 2008 (*T*1) and follow-up in 2011 (*T*2; *N* = 3044). Mean age at *T*1 was 62.9 years (40–93; SD = 11.6), 48.7% were female and 35.7% of participants lived in former East Germany. Assessment year 2008 was used as *T*1 as this is the only year in which the German Ageing Survey assessed age stereotypes and personal values.

### Dropout analyses

For dropout analyses, we conducted independent sample t-tests and calculated the effect size Hedges’ *g*, which takes unequal sample sizes into account. Hedge’s *g* = .2 can be considered a small effect, *g* = .5 a medium and *g* > .8 a large effect (Cohen 1988). Analyses of *T*1 variables showed that participants were more likely to participate in 2011 when they were already part of the panel (*t*(6044.01) = − 12.73, *p* < .001; *g* = .32). Participants dropping out between 2008 and 2011 were significantly older (*t*(5297.27) = 3.53, *p* < .001; *g* = .09) and less educated (*t*(5586.6) = − 11.81, *p* < .001; *g* = .31) than those who continued to take part in the study. Drop-outs reported worse physical function (*t*(4923.56) = − 6.27, *p* < .001; *g* = .17) and associated their own ageing more with social losses (*t*(5247.90) = 3.25, *p* = .01; *g* = .09) and less with ongoing development (*t*(5123.28) = − 8.46, *p* < .001; *g* = .22). They also reported less gain-related age stereotypes (*t*(5330.80) = − 6.86, *p* < .001; *g* = .18). For values, people for whom conservation and self-enhancement values were relatively important were more likely to drop out (*t*(6002) = 4.91, *p* < .001; *g* = .13 and *t*(5356.24) = 6.96, *p* < .001; *g* = .18, respectively). Those with a value priority of openness to change and self-transcendence were more likely to participate in the second measurement in 2011 (*t*(5359.88) = − 4.64, *p* < .001; *g* = .12 and *t*(6005) = − 8.39, *p* < .001; *g* = .22, respectively). Overall, the effects due to sample attrition were small.

### Measures

#### Self-perceptions of ageing (SPA)

We used two subscales of the AgeCog scales (Steverink et al. 2001; Wurm et al. 2007) to measure SPA. The social losses subscale refers to the perception of ageing as accompanied by loss of social status. One example item is “Ageing means to me that I am less respected”. Reliability for this subscale is Cronbach’s *α* = .75. The second subscale, ongoing development, refers to ageing as a time of continuing psychological growth (e.g. “Ageing means to me that I continue to make plans”). Reliability is good with Cronbach’s *α* = .82. Each subscale contains four items with a 4-point Likert-scale from 1 “definitely true” to 4 “definitely false”. For the analysis, scores were reverse coded and then averaged for the separate subscales, so that a higher score in AgeCog social losses indicates a higher notion of ageing being accompanied by social losses (loss-related SPA), and a higher score in AgeCog ongoing development indicates stronger perception of ageing as a time of ongoing development (gain-related SPA).

#### Age stereotypes (AS)

In the 2008-wave, the DEAS additionally used an adapted form of the AgeCog scales to measure age stereotypes. The items and scaling procedure were the same as for SPA, except for the item-stem which began with “Ageing means to *most people*…” instead of “Ageing means to *me*…”. These scales showed satisfactory internal consistency (Age stereotype social losses: Cronbach’s *α* = .78; Age stereotype ongoing development: Cronbach’s *α* = .73).

#### Personal values

Values were measured with the 21-item Portrait Values Questionnaire (Schwartz 2003). The items consist of short descriptions of a person gender-matched to the respondent. “Thinking up new ideas and being creative is important to her. She likes to do things in her own original way” is an example of the openness to change value. Participants indicated the extent to which they considered the described person to be similar to themselves on a Likert-scale ranging from 1 (not at all like me) to 6 (very much like me). Scores for the openness to change, self-enhancement, conservation and self-transcendence values were averaged over the corresponding items. Cronbach’s *α* are .71, .71, .73, and .70, respectively. We subtracted each respondent’s mean score across all values from each single value, as proposed by Schwartz (1992), thus correcting for interindividual differences in scale use tendencies and creating scores for value priorities. Thus, the score for each value represents not its absolute, but *relative* importance compared to the other values in the value system of each individual. For instance, a score of 2 would signify that this value is of relatively higher importance to a person than other values, a score of − 1 would signify that other values are more important.

#### Covariates

Since the DEAS is stratified by age, gender and place of residence (former East or West Germany), we used these covariates in all analyses. We also controlled for level of education according to the International Standard Classification of EDucation (ISCED; low (without completed vocational training), medium (with completed vocational training and/or high school Diploma) and high education (e.g. graduation from a technical school, vocational academy, school of business administration, or university) (Unesco 1997), as these variables are related to SPA in the DEAS sample (Wurm and Huxhold 2012). To control for sample selection, we added a covariate indicating whether a person belongs to the longitudinal part of the DEAS or the newly drawn subsample in 2008. As previous studies showed that SPA are interrelated with physical function (e.g. Sargent-Cox et al. 2012), the analyses were additionally controlled for the 10-item physical functioning subscale of the SF-36 Health Survey (Bullinger and Kirchberger 1998). Participants rated the extent of their limitations in everyday tasks on a three-point scale (not limited, limited to some extent, limited). Scores were transformed to a scale ranging from 1 to 100 with a higher score indicating better physical functioning (Cronbach’s *α* = .93).

### Analytical procedure

We used SPSS 25 for descriptive statistics, correlations, and dropout analyses. The hypotheses were tested via multiple regression analyses using Mplus 8 with robust Full Information Maximum Likelihood Estimation. The benefit of this method is that it uses all available data for the estimation, including the data of persons missing one measurement occasion, thus reducing potential bias caused by missing data. For each of the two SPA domains, we conducted two separate regression analyses to investigate which differential aspects of agentic (openness to change and self-enhancement) and communal (conservation and self-transcendence) value priorities would predict gain- and loss-related SPA. This resulted in four regression models.

In each analysis, we controlled for covariates, the dependent variable (SPA) at *T*1 and the corresponding domain-matched age stereotype. All predictors were *z*-standardized before the analyses to account for different scaling in the measures.

## Results

### Descriptive analyses

Table 1 gives an overview of sample characteristics of baseline variables at *T*1. Overall, age stereotypes were more negative than SPA: Participants perceived the ageing of most other people (age stereotypes) as significantly associated with more social losses (*t*(6019) = − 42.80, *p* < .001; Cohen’s *d* = .58) and less psychological gains (*t* (6022) = − 22.58, *p* < .001; Cohen’s *d* = .29) compared to their own ageing (SPA). Still, participants associated both their own ageing and the ageing of most other people more strongly with gains than losses as reflected in higher means for both gain-related SPA (*M* = 2.88; SD = 0.61) and age stereotypes (*M* = 2.71; SD = 0.58) than loss-related SPA (*M* = 1.86; SD = 0.59) and age stereotypes (*M* = 2.23; SD = 0.68). Concerning value priorities, self-transcendence (*M* = 0.84; SD = 0.54) and conservation values (*M* = 0.17; SD = 0.64) were considered relatively more important than openness (*M* = − 0.24; SD = 0.60) and self-enhancement values (*M* = − 0.92; SD = 0.71). A table with bivariate correlations of the study variables can be found in the Appendix (Table 3).

**Table 1:** Descriptive statistics for study variables in 2008 (*T*1)

	M or %	SD	Range
Age	62.89	11.64	40–93
Gender (1 = female) (%)	48.66		
Panel (1 = yes) (%)	27.05		
Region (1 = former East Germany) (%)	35.67		
Education (1 = low, 2 = middle, 3 = high)	2.27	0.62	1–3
Physical function	84.13	22.24	0–100
SPA social losses	1.86	0.59	1–4
SPA ongoing development	2.88	0.61	1–4
Age stereotype social losses	2.23	0.68	1–4
Age stereotype ongoing development	2.71	0.58	1–4
Value priority of openness to change	−0 .24	0.60	− 2.5 to 2.48
Value priority of self-enhancement	− 0.92	0.71	− 3.25 to 2.24
Value priority of conservation	0.17	0.64	− 2.55 to 3.05
Value priority of self-transcendence	0.84	0.54	− 1.19 to 2.76

*SPA* self-perceptions of ageing

### Age stereotypes and SPA

We used multiple regression analyses to investigate the importance of value priorities beyond age stereotypes and other covariates at *T*1 for SPA at *T*2. Table 2 shows the coefficients of the regression analyses based on z-standardized predictors. Loss- and gain-related SPA at *T*2 were most strongly predicted by the corresponding SPA at *T*1 (*B* = .30, SE = .01, *p* < .001 for social losses and *B* = .31, SE = .01, *p* < .001 for ongoing development), indicating that SPA are rather stable over the time span of 3 years. Domain-matched age stereotypes significantly predicted SPA at *T*2 in all analyses, showing in line with previous studies that age stereotypes on social losses contribute to SPA social losses (*B* = .02, SE = .02, *p* < .001 for both agentic and communal values), whereas age stereotypes on ongoing development significantly predicted SPA in this domain (*B* = .03, SE = .01, *p* < .01 for both analyses).

**Table 2:** Regression models of SPA related to social losses and ongoing development at *T*2

Predictors	DV = SPA social losses *T*2		DV = SPA ongoing development *T*2	
	*B* (SE)	*B* (SE)	*B* (SE)	*B* (SE)
Loss-related SPA	0.30 (.01)***	0.30 (.01)***		
Gain-related SPA			0.31 (.01)***	0.31 (.01)***
Age	− 0.05 (.01)***	0.04 (.01)***	− 0.10 (.01)***	− 0.09 (.01)***
Gender (1 = female)	0.000 (.01)	0.01 (.01)	0.01 (.01)	0.005 (.009)
Panel (1 = yes)	− 0.03 (.02)	− 0.03 (.02)	0.01 (.02)	0.003 (.01)
Region (1 = former East Germany)	0.003 (.01)	− 0.001 (.01)	− 0.04 (.01)***	− 0.03 (.01)***
Education (1 = low, 2 = middle, 3 = high)	− 0.04 (.01)***	− 0.03** (.01)	0.04 (.01)***	0.03 (.01)**
Physical function	− 0.04 (.01)**	− 0.04 (.01)**	0.05 (.01)***	0.05 (.01)***
Age stereotype social losses	0.02 (.01)*	0.02 (.01)*		
Age stereotype ongoing development			0.03 (.01)**	0.03 (.01)**
Value priority of openness to change	− 0.001 (.01)		0.03 (.01)***	
Value priority of self-enhancement	0.03 (.01)***		− 0.01 (.01)	
Value priority of conservation		0.004 (.01)		− 0.04 (.01)***
Value priority of self-transcendence		− 0.04 (.01)***		0.02 (.01)*
	*R*^2^ = 0.341***	*R*^2^ = 0.342***	*R*^2^ = 0.461***	*R*^2^ = 0.462***

*DV* dependent variable, *SPA* self-perceptions of ageing

*p < .05; **p < .01; ***p ≤ .001

### Agentic values and SPA

As for the role of agentic values, we hypothesized that a person for whom *openness to change* is relatively important perceives his or her ageing as related to more ongoing development at follow-up. As expected, openness to change predicted this SPA facet (*B* = .03, SE = .01, *p* < .001), yet was, also in line with the hypothesis, not associated with SPA related to social losses. The second hypothesis was that prioritizing *self*-*enhancement* was associated with more SPA as related to social losses at follow-up. This hypothesis was also supported (*B* = .03, SE = .01, *p* < .01). In addition, self-enhancement was unrelated to the perception of ongoing development. These results indicate domain-specific effects of both agentic values on SPA.

### Communal values and SPA

We tested the impact of communal value priorities on SPA in a separate set of regression analyses. Corroborating our hypotheses, communal value priorities differentially predicted domain-specific SPA: The more a person prioritized *conservation*, the less they perceived ageing to be associated with ongoing development 3 years later (*B* = − .04, SE = .01, *p* < .001). Conservation value priority was unrelated to the perception of social losses. *Self*-*transcendence* value priority, on the other hand, was a significant predictor of both domains: It predicted less loss-related SPA (*B* = − .04, SE = .01, *p* < .001) and more gain-related SPA (*B* = .02, SE = .01, *p* = .032) at follow-up.

## Discussion

The goal of this study was to examine whether the priority of certain personal values plays a role for SPA beyond the impact of domain-matched age stereotypes. We focused on two domains of self-perceptions of age-related gains and losses: psychological development (making new plans and realizing ideas) and social losses (receiving less respect and feeling lonelier). We hypothesized differential effects of the four value priorities of openness to change, self-enhancement, conservation and self-transcendence on gain- and loss-related SPA. Results supported the hypotheses. All four value priorities emerged as independent predictors of domain-specific SPA despite controlling for domain-specific age stereotypes and other covariates, thus providing first evidence that value priorities do play a role for SPA.

A higher value priority of openness to change predicted higher gain-related SPA, that is, a more positive perception of ageing as a time of new opportunities and psychological growth at follow-up. This might be due to a generally more optimistic focus on existing opportunities for development in older age and thus might point to better adjustment capability. People for whom openness to change is especially important might more readily accept and integrate changing capacities. This value priority might also facilitate a shift of focus from unattainable to more reachable goals. On the contrary, higher priority of self-enhancement at baseline predicted the perception of more social losses at follow-up. This means that if power and achievement was most important to a person, he or she perceived ageing more as associated with loss of respect, boredom and loneliness. One explanation might be that individuals who define themselves by their socially acknowledged achievement and power over others are especially prone to perceiving age-related loss of control in a negative way. Opportunities for social appreciation are likely to decline with age, when, for example, work-related success is no longer available as a source of validation. Value priority of achievement might generally be associated with increased stress and depression, as Hanel and Wolfradt (2016) found in a student sample. This value-associated stress might accumulate over the life span and result in a loss-related evaluation of the ageing process.

People with a priority of the value conservation reported less gain-related SPA after 3 years; i.e. someone for whom it is relatively important to stick to tradition and maintain the status quo perceives ageing less as a time of new plans and activities. Conservation implies a stronger focus on the maintenance of what has already been achieved and is thus less a driver for new plans. On the other hand, a value priority of conservation might be a sign of limited resources. According to Schwartz (2012), conservation is a value that addresses coping with uncertainties and anxiety in a rather passive way through avoiding conflict and maintaining the current order; this preoccupation might reflect a lack of psychological resources that could be needed for successful adaption to age-related changes (Schwartz 2010).

As for self-transcendence, results indicate that individuals who prioritize benevolence and universalism perceive their own ageing as associated with more psychological gains as well as less social losses at follow-up. This result recalls Lars Tornstam’s gerotranscendence theory, in which he defines gerotranscendence as “a shift in meta-perspective, from a materialistic and pragmatic view of the world to a more cosmic and transcendent one” (Tornstam 1997, p. 143). This shift in focus away from an egocentric value orientation to a more universal view might be indicative of successful adaptation to the ageing process, integrating both age-related gains and losses in a positive way.

Taken together, this study supports and extends findings on the impact of personality traits on SPA by providing first evidence for the role of personal value priority. It thus seems of benefit to look at personality not only from a “Big Five-perspective”, but to also include other personality aspects, such as values, in the discussion of SPA. A special emphasis lies on the role of self-transcendence values as a probable facilitating factor in successful adaption to age-related changes that may be reflected in more gain-related and less loss-related SPA in the domains of ongoing development and social losses. It seems to be beneficial for older individuals to shift their focus from pursuing self-centred goals to a more universal concern with the well-being of others—distant or close.

### Limitations and strengths

The study has some limitations which need to be considered. First of all, data came from a large German national panel of community-dwelling adults that is affected by selective sample attrition: participants dropping out were less healthy, less educated and older than the participants who could be followed longitudinally and also differed in their SPA and values. These effects were, however, small in size. Though the use of Full Information Maximum Likelihood estimation does not solve the issue of selective attrition, it at least adds more information to the estimation than full-case analyses would, thereby reducing potential bias. Another limitation was that values and age stereotypes where only asked for in the 2008 wave of the German Ageing Survey. Thus, we were not able to model the change in value priorities and age stereotypes over time, which might have provided additional insights into the relationship between values, age stereotypes and SPA. Additionally, it would have been interesting to model the information added by values above personality traits, but traits have not been assessed in the survey. This question should thus be addressed in future studies. Furthermore, the effects of value priorities are quite small. This is most likely due to the relative stability of SPA. SPA generally do not change much, unless there is a critical life event like, for example, a cardiovascular event (Wurm et al. 2019). Due to this stability, only minor effects on SPA can be expected. Furthermore, age stereotypes are well supported predictors of SPA and we were still able to find a comparable impact of personal values on SPA beyond these stereotypes. Further strengths of our study lie in the large sample and the longitudinal design as well as the fresh insights gained by investigating the role of personal values for SPA for the first time. Also, we investigated SPA not on a global but domain-specific level, as is increasingly called for in the literature (e.g. Kornadt et al. 2019b). This allows for a more detailed understanding of associations between the different value priorities and gain- and loss-related domains of SPA.

### Implications and future research

The results of the study suggest that personal value priorities partly account for the multidimensional nature of self-perceptions of ageing: what people find most important shapes which aspects of the ageing process they focus their attention on. Future research should thus further investigate the role of values for other domains of SPA such as physical losses or the domains of family and work (e.g. Kornadt and Rothermund 2015). Also, research could investigate mechanisms to reduce anxiety and worries in individuals who prioritize the deficiency-related values conservation and self-enhancement, thus freeing resources needed to focus on age-related gains. It is also worth further exploring the pathways through which values might lead to actual negative experience with ageing and thus negative SPA. One way might be behaviour: for example, people for whom power is relatively important tend to engage in unhealthy behaviour such as smoking, unhealthy eating habits and irregular exercise (Honka et al. 2019). Future studies should thus consider different mechanisms through which personal values affect age-related experiences.

## Conclusions

In this study, we were able to provide first evidence for the role of personal value priorities for two domains of SPA over a 3-year period: ageing as ongoing personal development and ageing as related to loss in social status. This finding expands on previous research on longitudinal associations of the Big Five personality traits and SPA by adding another aspect of personality to the debate. We found all four value priorities to be related to gain- and loss-related SPA in specific and meaningful ways: openness to change predicted more and conservation less gain-related SPA, whereas self-enhancement predicted more loss-related SPA over time. Self-transcendence predicted more gain- and less loss-related SPA, thus reflecting an important resource for overall positive SPA. Research in gerontology should more often consider personal values when investigating the attitudes of older persons, since what is most important to them determines which part of the ageing process attracts their attention and whether they perceive ageing as threat or chance.

## Appendix

See Table 3.

**Table 3:** Bivariate correlations of study variables

	1	2	3	4	5	6	7	8	9	10	11	12	13	14	15
1. Age	–														
2. Gender	− 0.09***	–													
3. Educ.	− 0.16***	− 0.20***	–												
4. PF	− 0.36***	− 0.07***	0.19***	–											
5. Panel	0.15***	0.004	0.03*	− 0.03**	–										
6. Place	− 0.01	0.02	0.10***	− 0.07***	− 0.01	–									
7. AS SL	− 0.08***	0.02	− 0.02	− 0.10***	− 0.02	− 0.11***	–								
8. AS OD	− 0.16***	− 0.004	0.13***	0.26***	0.01	− 0.01	0.41***	–							
9. SPA SL *T*1	0.05***	− 0.02	− 0.13***	− 0.20***	− 0.02	− 0.004	− 0.45***	− 0.28***	–						
10. SPA OD *T*1	− 0.26***	0.02	0.20***	0.34***	− 0.003	− 0.10***	− 0.17***	0.53***	− 0.41***	–					
11. O	− 0.19***	− 0.05***	0.12***	0.16***	0.02	− 0.10***	0.01	0.20**	− 0.08***	0.33***	–				
12. SE	− 0.17***	− 0.17***	0.10***	0.09***	− 0.06***	− 0.03*	0.03*	− 0.02	0.06***	− 0.003	− 0.01	–			
13. C	0.33***	0.03*	− 0.22***	− 0.19***	0.005	0.13***	− 0.07***	− 0.16***	0.10****	− 0.33***	− 0.74***	− 0.35***	–		
14. ST	− 0.04**	0.21***	0.05**	− 0.02	0.04**	− 0.02	0.05**	− 0.01	− 0.11***	0.04**	− 0.24***	− 0.54***	− 0.08***	–	
15. SPA SL *T*2	0.08***	− 0.004	− 0.12***	− 0.15***	− 0.03	− 0.01	0.25***	− 0.17***	0.54***	− 0.31***	− 0.05**	0.06**	0.09***	− 0.14***	–
16. SPA OD *T*2	− 0.28***	0.03	0.16***	0.26***	− 0.04*	− 0.10***	− 0.03	0.31***	− 0.26***	0.60***	0.24***	0.02	− 0.28***	0.06**	− 0.38***

*T*1 = baseline (2008); *T*2 = follow-up (2011), *Educ.* education, *PF* physical functioning, *Place* place of residence (former East/West Germany), *AS* age stereotype, *SL* social losses, *OD* ongoing development, *SPA* self-perceptions of ageing, *O* value priority of openness to change, *SE* value priority of self-enhancement, *C* value priority of conservation, *ST* value priority of self-transcendence

**p* < .05; ***p* < .01; ****p* ≤ .001
